# Transcriptome Signatures for Cognitive Resilience Among Individuals with Pathologically Confirmed Alzheimer Disease

**DOI:** 10.1002/alz.090972

**Published:** 2025-01-03

**Authors:** Donghe Li, Xudong Han, Lindsay A. Farrer, Thor D. Stein, Gyungah R Jun

**Affiliations:** ^1^ Boston University Chobanian & Avedisian School of Medicine, Boston, MA USA; ^2^ Departments of Medicine (Biomedical Genetics), Neurology, Ophthalmology, Biostatistics, and Epidemiology, Boston University Schools of Medicine and Public Health, Boston, MA USA; ^3^ Department of Pathology and Laboratory Medicine, Boston University Chobanian & Avedisian School of Medicine, Boston, MA USA; ^4^ VA Boston Healthcare System, Boston, MA USA; ^5^ Department of Biostatistics, Boston University School of Public Health, Boston, MA USA; ^6^ Department of Ophthalmology, Boston University Chobanian & Avedisian School of Medicine, Boston, MA USA; ^7^ Department of Medicine (Biomedical Genetics), Boston University Chobanian & Avedisian School of Medicine, Boston, MA USA

## Abstract

**Background:**

The molecular mechanisms underlying individuals with neuropathologically confirmed Alzheimer disease (AD) but who were cognitively healthy prior to death (i.e., cognitively resilient) remain largely unknown.

**Method:**

We evaluated brain‐derived RNA sequencing data from 213 individuals with cognitive resilient and 383 symptomatic AD cases. This data included newly generated sequences from Boston University Alzheimer’s Disease Research Center (BUADRC) participants, and existing data from the Framingham Heart Study (FHS), Religious Orders Study, Rush Memory and Aging Project (ROSMAP), and the Mount Sinai Brain Bank (MSBB). Differential gene expression analyses between cognitive resilient and symptomatic AD were conducted in each dataset and then the results were combined by meta‐analysis. Differentially expressed genes (DEGs) at the transcriptome‐wide significance (TWS) level (P<10^‐6^) in meta‐analysis and nominally significant (P<0.05) in at least two datasets were included in subsequent analyses of pathways and associations with cognitive and neuropathological traits.

**Result:**

We identified 42 TWS genes, including 17 that were meta‐analysis P<5 × 10^‐8^. The two most significantly DEGs in meta‐analysis, *ADAMTS2* (Log2 fold change [Log2FC] = 0.45, P = 2.25 × 10^‐14^) and *SCGN* (Log2FC = 0.57, P = 1.84 × 10^‐11^), were both up‐regulated in symptomatic AD brains. Significant pathways involving alpha‐amino acid catabolic process and chemical synaptic transmission modulation were identified. These genes exhibited distinct patterns of association with domain‐specific cognitive tests, which the most significant association was found with *SMPX* expression for memory (beta = ‐0.08, P = 0.002). Regarding neuropathological traits, *IL2RG* expression was the most significant association with pTau181 (beta = ‐3.56, P = 0.002). Of the two most significant DEGs, *ADMTS* expression was associated with memory (beta = ‐0.013, P = 0.002) and pTau231 (beta = 0.008, P = 0.005), while *SCGN* expression was associated with language (beta = ‐0.1, P = 0.025) and executive function (beta = ‐0.1, P = 0.02).

**Conclusion:**

We identified multiple DEGs for cognitive resilience, several of which were also associated with measures of cognitive performance prior to death and AD‐related neuropathological traits at autopsy, thus providing important insights into resilience mechanisms and strategies for delaying clinical symptoms of AD. Notably, *ADAMTS2* was the top‐ranked DEG in a study conducted in brains from African American AD cases and controls (see Logue et al. abstract for this meeting).

